# Health Department Problem

**Published:** 1907-02

**Authors:** 


### Health Department Problem.

HEALTH COMMISSIONER WENDE has cancelled the permit under which a slovenly butcher conducted a slaughter house. This was done after two hearings and a warning. The place maintained by this butcher was reported by Dr. Wende's inspector as "filthy."

Much time has been spent, since the slaughter house scandal, in searching out the unsanitary places in Buffalo in connection with its meat supply, and everything has been fairly well cleaned up.

There are other things which might be considered in connection with public health, matters which have been corrected by the health departments of other cities. One is flat wheels on the street cars which rip-slam-bang their riotous way through the residential streets making days disturbing and nights hideous.

The nerves of passengers would be relieved of rather high tension if attention were paid to the screeching of metal bound street car doors and the squawking of register rods.

Siren whistles with their unholy lost-soul wails, the assinine whistle salutes of tugs and boats which greet the arrival of anything new or novel in the marine line; the yells, the shouts, the curses and the "get-aps" and "whoa-a-a-as" and the banging of ash cans by the drivers and workers on the wagons of the Buffalo Sanitary Company which has the cleaning contracts; these are all matters which affect the public health and ought to be eliminated.

---

THE growth of the average finger nail is computed to be one-thirty-second of an inch a week, or a little more than an inch and a half a year. The finger nails, according to "Popular Science Siftings," are said to grow faster in the summer than in the winter. The nail on the middle finger grows faster than any of the other nails, and that on the thumb grows slowest. It is also said that the nails on the right hand grow faster than those on the left hand. According to the rate of growth stated, the average time taken for each finger nail to grow its full length is about four and a half months, and at this rate a man seventy years old would have renewed his nails one hundred and eighty-six times. Taking the length of each nail as half an inch, he would have grown seven feet nine inches of nail on each finger, and on all his fingers and thumbs an aggregate length of seventy-seven and a half feet.

---

IT was a singularly beautiful law of nature, observes *The Tribune*, which Mendeléeff, the Russian chemist, whose death is announced, was the first to observe. He noticed that when the elements were grouped on the sole principle of similarity of qualities a remarkable relationship also existed between the atomic weights of the members of a family. It was on the strength of that strange fact that Sir William Ramsay, after terrestrial helium and argon had been found, predicted the discovery of another primary substance resembling them and possessing characteristics which were distinctly specified. The forecast, it will be remembered, had a triple verification at Sir William's own hands. Within a surprisingly short time he added neon, krypton and xenon to the list of prevously recognised elements, and they all fitted with comparative exactness into the proper places in Mendeléeff's table.

---

The offices of this Journal have been removed from 284 Franklin St., where they had been established for nearly twenty years, to 238 Delaware Avenue, corner Chippewa Street, in the Delaware Court Building. All communications relating either to the editorial or business management of the Journal should be sent to the new address.

